# The systemic inflammation response index as a significant predictor of short-term adverse outcomes in acute decompensated heart failure patients: a cohort study from Southern China

**DOI:** 10.3389/fendo.2024.1444663

**Published:** 2024-12-23

**Authors:** Lin Xie, Qun Wang, Hengcheng Lu, Maobin Kuang, Shiming He, Guobo Xie, Guotai Sheng, Shuhua Zhang, Wei Wang, Yang Zou

**Affiliations:** ^1^ Jiangxi Cardiovascular Research Institute, Jiangxi Provincial People’s Hospital, The First Affiliated Hospital of Nanchang Medical College, Nanchang, Jiangxi, China; ^2^ Jiangxi Medical College, Nanchang University, Nanchang, Jiangxi, China; ^3^ Department of Cardiology, Jiangxi Provincial People’s Hospital, The First Affiliated Hospital of Nanchang Medical College, Nanchang, Jiangxi, China

**Keywords:** ADHF, acute decompensated heart failure, SIRI, systemic inflammation response index, inflammatory marker

## Abstract

**Objective:**

The deterioration of acute decompensated heart failure (ADHF) is associated with abnormal activation of inflammatory pathways. This study aims to evaluate the impact and predictive value of a novel inflammatory marker, the systemic inflammation response index (SIRI), on short-term adverse outcomes in ADHF patients.

**Methods:**

This retrospective cohort study included 1,448 ADHF patients from Jiangxi Provincial People’s Hospital between 2019-2022. SIRI was calculated using the formula: (neutrophil count × monocyte count)/lymphocyte count. In the correlation analysis, the study outcome was the 30-day mortality in patients with ADHF. Cox regression analysis and receiver operating characteristic curves were employed to investigate the risk assessment and predictive value of the SIRI for 30-day mortality in ADHF patients. Finally, we also exploratively assessed the mediation effect of nutritional factors (albumin: Alb, total cholesterol: TC, and lymphocyte count) on the association between SIRI and 30-day mortality in ADHF patients.

**Results:**

During the 30-day follow-up, 53 deaths were recorded. Mortality rates across SIRI tertiles were 0.62%, 2.07%, and 8.28%, respectively. There was a significant linear positive correlation between SIRI and 30-day mortality in ADHF patients (HR: 1.21; *P* for non-linearity = 0.113). Additionally, compared to ADHF patients with low SIRI, those with high SIRI had a 685% increased risk of 30-day mortality (HR: 7.85). Furthermore, receiver operating characteristic curve analysis demonstrated that SIRI significantly improved the predictive value for 30-day mortality in ADHF patients compared to neutrophil count, monocyte count, and lymphocyte count alone (AUC: neutrophil count 0.7633, monocyte count 0.6835, lymphocyte count 0.7356, SIRI 0.8237; all DeLong *P*<0.05). Mediation analyses indicated that, except for lymphocyte count, both Alb and TC had significant indirect effects on the SIRI-related 30-day mortality in ADHF patients; Specifically, Alb accounted for approximately 24.46% of the mediation effect, while TC accounted for approximately 13.35%.

**Conclusion:**

This cohort study based on a Southern Chinese population demonstrates a significant linear positive correlation between SIRI and 30-day mortality in ADHF patients, highlighting its substantial predictive value. Incorporating SIRI into the monitoring regimen of ADHF patients may be crucial for preventing further disease progression.

## Background

Current guidelines define acute decompensated heart failure (ADHF) as the presence of new or worsening symptoms and signs of heart failure (HF), where “decompensated” indicates an urgent need for therapeutic intervention to alleviate these symptoms and signs (1–3). Despite recent advances in our understanding, the pathophysiology of ADHF remains poorly elucidated, resulting in limited treatment options and making ADHF one of the most challenging inpatient conditions to manage effectively (1, 4–6). Severe adverse events are common shortly after ADHF onset, with 30-day mortality and rehospitalization rates reported at approximately 10% and 25%, respectively (1, 4, 7–9). These outcomes substantially impact patients and their families, imposing a significant burden on healthcare systems. Thus, early identification of key factors influencing ADHF deterioration is essential.

Although the exact pathophysiology of ADHF is not fully understood yet (1, 4), it should be closely related to the state of physical stress, which is prevalent in many acute episodes of cardiovascular disease (10, 11). Previous studies have shown that multiple inflammatory factors are activated during ADHF attacks and further perpetuate the inflammatory state even after each attack subsides (12–14). Notably, elevated levels of inflammatory markers in HF patients often precede neurohormonal biomarkers [e.g., N-Terminal Pro-Brain Natriuretic Peptide (NT-pro BNP)] and are closely associated with disease severity and prognosis (15, 16). These findings suggest that inflammatory factors may serve as sensitive prognostic indicators for ADHF patients, emphasizing the importance of early inflammatory assessment.

Recently, the systemic inflammation response index (SIRI), combining neutrophil, monocyte, and lymphocyte counts, has collected attention as an inflammatory marker. Studies have demonstrated that SIRI can independently predict the prognosis of various cancers (17–21) and cardiovascular diseases (22–28). Additionally, SIRI has been used to evaluate the risk of chronic kidney disease (29, 30), psoriasis (31), deep vein thrombosis (32), osteoporosis (33), and periodontitis (34), as well as activity risks of rheumatoid arthritis (35). Currently, however, limited research exists on the association between the SIRI and HF prognosis. Completed studies have primarily focused on chronic HF and populations with ischemic HF following percutaneous coronary intervention (PCI) (36–38), lacking relevant research data in patients with ADHF. Given the significant adverse outcomes associated with ADHF, this study aims to determine the impact and predictive value of SIRI on short-term adverse outcomes in ADHF patients through a retrospective cohort study in Jiangxi, China.

## Methods

### Study population and design

This retrospective cohort study consecutively enrolled 1,790 ADHF patients admitted to Jiangxi Provincial People’s Hospital from January 2019 to December 2022. The diagnosis of ADHF was based on the latest available European Society of Cardiology guidelines for the diagnosis and treatment of acute and chronic HF. The exclusion criteria for the study population were as follows: (i) To minimize the potential impact of water-sodium retention, we excluded patients with cirrhosis, uremia and those undergoing dialysis treatment (n=122); (ii) patients with malignancies were excluded due to their impact on survival (n=73); (iii) patients who underwent PCI within the past three months were excluded because reperfusion therapy typically influences short-term outcomes (n=42); (iv) patients under 18 years of age (n=12); (v) pregnant participants (n=1); (vi) patients with pacemakers were excluded due to their expected lack of autonomic heart rate control (n=63); (vii) patients with missing SIRI data were also excluded (n=29). This study was conducted in accordance with the principles of the Declaration of Helsinki, and the use of patient data was explained to and authorized by the patients and their families. The study received approval from the Ethics Committee of Jiangxi Provincial People’s Hospital (IRB: 2024-01). The research strictly followed the STROBE guidelines (**Supplementary Table 1**).

### Data collection

Baseline data were extracted from the hospital’s electronic medical record system by two trained scientific staff. These data included blood pressure measured at admission [using an Omron automatic blood pressure monitor (HBP-1300) in a quiet environment or at the bedside], hematological parameters, echocardiographic parameters, demographic data (gender and age), heart function assessment [New York Heart Association (NYHA) classification)], comorbidity information (including hypertension, diabetes, stroke, and coronary artery disease), and information on medications received during hospitalization [Including sodium-dependent glucose transporters 2 (SGTL-2), digitalis, beta-blockers, diuretic, angiotensin receptor inhibitors (ARB)/angiotensin-converting enzyme inhibitors (ACEI)/angiotensin receptor neprilysin inhibitors (ARNI)]. The determination of comorbidities also referenced the patient’s medical records, medication information, and auxiliary examination results during hospitalization.

Laboratory parameters were measured within 24 hours of patient admission. It should be noted that blood count [white blood cell count (WBC), neutrophil count, lymphocyte count, monocyte count, red blood cell count (RBC), hemoglobin (HGB), platelet count (PLT)], albumin (Alb), creatinine (Cr), blood urea nitrogen (BUN), and N-Terminal Pro-Brain Natriuretic Peptide (NT-proBMP) were usually measured immediately on admission, whereas liver enzymes [alanine aminotransferase (ALT), aspartate aminotransferase (AST)] and fasting blood glucose (FPG), lipids [total cholesterol (TC), triglycerides (TG), low-density lipoprotein cholesterol (LDL-C), high-density lipoprotein cholesterol (HDL-C)] were measured by venous blood sampling either on admission in a fasting state or early in the morning of the second day after admission.

Blood samples were drawn by trained nurses using standardized needles and blood collection tubes, and then sent to the Jiangxi Provincial People’s Hospital Laboratory Center for specimen eligibility assessment. The unique identification number and test items of each patient were re-verified. After confirming that the blood sample met the testing requirements, professional inspectors will use the Sysmex XN-3000 (Sysmex Co, Kobe, Japan) automatic blood analyzer and HITACHI LAbOSPECT 008 (Hitachi High-Tech Co, Tokyo, Japan) automatic biochemical analyzer performed blood cell analysis and biochemical analysis. Notably, the Sysmex XN-3000 and HITACHI LAbOSPECT 008 autoanalyzers have high precision, good linear ranges, reliable clinical reportable ranges and reference intervals, and were able to meet the clinical testing requirements very well (39, 40). According to the data provided by the manufacturer, the analytical variation coefficients of blood cell count, FPG, BUN, Cr, UA, lipids, liver enzymes, Alb and NT-proBNP were ≤6%, ≤5%, ≤4%, ≤5%, ≤4%, ≤4%, ≤5%, ≤4% and ≤ 8%, respectively.

For laboratory quality control, our center conducted daily sample testing with the results consistently within control limits. The accuracy of the laboratory’s results was assessed through participation in national and provincial inter-room quality assessments and daily in-house quality control. Inter-laboratory quality assessments are performed 5-10 times annually, and in-house quality control is conducted daily; if any results are out of control, the cause needs to be analyzed and the problem solved to ensure that samples are tested under control.

### SIRI calculation

The Systemic Inflammation Response Index (SIRI) was calculated as follows: (neutrophil count × monocyte count)/lymphocyte count (17).

### Study outcomes

The primary outcome of this study was the incidence of all-cause mortality within 30 days following the onset of ADHF in the subjects. For all ADHF patients in the cohort, the admission time was set as the start of follow-up, and 30-day survival status was obtained by trained medical staff via text messages, phone calls, and face-to-face follow-ups in outpatient/inpatient settings.

### Statistical analysis

Subjects were divided into three groups based on their SIRI levels (low, moderate, high) to display and compare baseline characteristics. Data were presented according to their type and distribution characteristics [reported as mean (standard deviation), median (interquartile range), or frequency (percentage)] and appropriate statistical methods (one-way ANOVA, Kruskal-Wallis H test, or chi-square test) were used for comparisons between groups.

Considering that the endpoint event of the current study is a dichotomous variable containing survival data, the Cox proportional hazards models were first considered for association analysis. Before constructing the Cox regression model, we first performed Kaplan-Meier analysis to evaluate the survival status across the three SIRI groups, and plotted the Schoenfeld residual plot of SIRI changes over time (**Supplementary Figure 1**) to verify whether the Cox regression model used in the current analysis complied with the proportional hazards assumption (41). In addition, we assessed the covariance between the independent variables and the covariates through variance inflation factors, and covariates with variance inflation factors greater than 5 will not be included in the multivariate-adjusted Cox regression models (42) (**Supplementary Table 2**). According to the STROBE guideline recommendations, four stepwise-adjusted Cox regression models were established to estimate hazard ratios (HRs) and 95% confidence intervals (CIs) (43). Model 1 was adjusted for basic information assessed at admission, including gender, age, hypertension, diabetes, stroke, CHD, NYHA classification, SBP, and DBP. Model 2 was further adjusted for left ventricular ejection fraction (LVEF), NT-proBNP, Cr, FPG, Alb, RBC, and PLT. The final model (Model 3) included additional adjustments for AST, TG, HDL-C, LDL-C, and BUN. Based on the final model, restricted cubic spline (RCS) model with four knots was applied to fit the relationship between SIRI and 30-day mortality in ADHF patients, with linear or non-linear associations were tested using the likelihood ratio test by comparing a model with only linear terms to a model with linear and cubic spline terms. For the selection of knots in RCS analysis, we followed the recommendations of Professor Harrell in the Regression Modeling Strategies book: When the number of knots is 4, the model fitting is improved, as it balances curve smoothness while avoiding the accuracy reduction associated with overfitting. When the sample size is larger, 5 knots is a better choice. For small samples (n<30) 3 nodes can be selected (44).

Subgroup analyses were conducted to test potential modification effects; stratified analyses were performed based on age [two groups: <65 years and ≥65 years, considering differences in population health status and HF susceptibility (45)], gender, LVEF, NYHA classification, and comorbidities. Interaction between stratification factors and SIRI was examined using the likelihood ratio test.

Receiver operating characteristic curve analysis was used to investigate the predictive value of SIRI and its components (neutrophil count, monocyte count, and lymphocyte count) for 30-day mortality in ADHF patients. The area under the curve (AUC), optimal threshold, sensitivity, and specificity were calculated. The significance of differences between AUCs was assessed using the DeLong test (46). In addition, we further investigated the predictive performance of adding SIRI to the baseline risk model (Including gender, age, hypertension, diabetes, stroke, CHD, NYHA classification, SBP, DBP) by calculating the C-index, Net Reclassification Improvement and Integrated Discrimination Improvement for quantifying and evaluating the ability of SIRI to improve the baseline risk prediction model.

Several sensitivity analyses were performed to test the robustness of the results: First, the final model included a quadratic term for age to account for potential non-linear associations between age and adverse outcomes (47). Second, considering the potential impact of multimorbidity on adverse outcomes (48), a subgroup excluding patients with three chronic diseases at baseline was analyzed. Third, the primary analysis was repeated after excluding deaths occurring within the first three days of follow-up (49). Fourth, it should be noted that there are still some variables with missing information in the current study (**Supplementary Table 3** shows the proportion of missing data in the study; **Supplementary Table 4** analyzes the baseline comparisons of missing and non-missing data, and the results showed that the missing data were randomized). To mitigate the impact of missing covariate data, missing values were estimated using the K-nearest neighbor interpolation (KNN) method, and the main analysis steps were repeated; It is worth mentioning that the KNN interpolation algorithm is a commonly used and effective method for filling in missing data, in addition to replacing the missing data with reasonable values as close as possible to the true values, the interpolation algorithm preserves the original data structure and avoids distorting the distribution of the interpolated variables (50, 51). Fifth, based on the published literature, it is clear that the SIRI can be used to assess the risk of chronic kidney disease, psoriasis, deep vein thrombosis, osteoporosis, periodontitis, as well as the risk of rheumatoid arthritis activity (29–35). Based on the specifics of this study, we further excluded patients with a history of chronic kidney disease, deep vein thrombosis, rheumatoid arthritis, and periodontitis and repeated the main analysis. Sixth, we further considered the potential impact of treatment factors in multivariate models.

All data analyses were conducted using R software version 4.2.1 and Empower(R) version 2.0, with significance evaluated using two-sided *P*-values, and a *P*-value < 0.05 was considered statistically significant.

## Results

### Baseline characteristics according to SIRI groups

**Figure 1:**
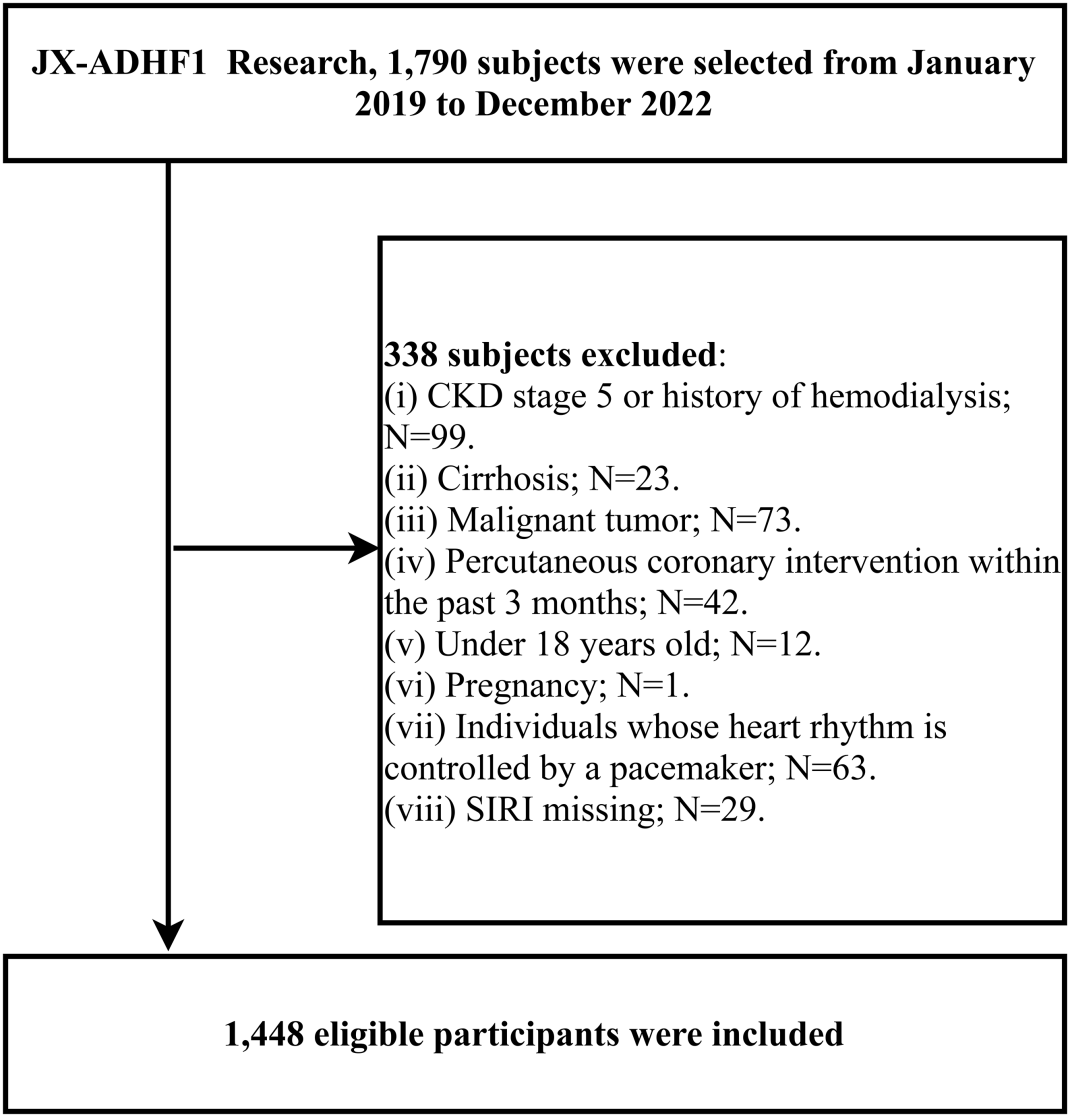
Flow chart for inclusion and exclusion of study participants.

**Figure 1** illustrates the study population’s selection process. Of the 1,448 ADHF patients included in the study, 832 were male and 616 were female, with an average age of 68 years. The baseline characteristics of ADHF patients, stratified by SIRI tertiles, were summarized in **Table 1**. Overall, compared to ADHF patients with low SIRI, those with high SIRI were more likely to be male, older, have diabetes, and be in NYHA class IV; They also had higher levels of WBC, neutrophil count, monocyte count, PLT, ALT, AST, Cr, BUN, FPG, and NT-proBNP, and lower levels of lymphocyte count, Alb, TC, HDL-C, and LDL-C. In addition, we summarized information on anti-heart failure medication during hospitalization in the study population according to SIRI tertile subgroups (**Table 1**); The results were summarized as follows: (1) There were no significant differences in treatment with SGLT-2 and diuretics among the three study populations. (2) ADHF patients with high SIRI had a relatively lower rate of using ACEI/ARB/ARNI and Beta-blockers, and a relatively higher rate of using Digitalis.

**Table 1:** Summary of baseline characteristics of the study population according to SIRI tertiles group.

	SIRI tertiles			*P*-value
	Low (0.14-1.31)	Moderate (1.31-2.78)	High (≥2.79)	
No. of subjects	483	482	483	
Age (years)	67.00 (57.00-75.50)	70.00 (61.25-80.00)	72.00 (64.00-80.00)	<0.001
Gender				<0.001
Female	252 (52.17%)	191 (39.63%)	173 (35.82%)	
Male	231 (47.83%)	291 (60.37%)	310 (64.18%)	
Comorbidities				
Hypertension (n,%)	182 (37.68%)	218 (45.23%)	199 (41.20%)	0.059
Diabetes (n,%)	93 (19.25%)	126 (26.14%)	151 (31.26%)	<0.001
Stroke (n,%)	69 (14.29%)	73 (15.15%)	89 (18.43%)	0.179
CHD (n,%)	130 (26.92%)	181 (37.55%)	138 (28.57%)	<0.001
NYHA classification (n,%)				<0.001
III	376 (77.85%)	355 (73.65%)	272 (56.31%)	
IV	107 (22.15%)	127 (26.35%)	211 (43.69%)	
SBP (mmHg)	127.20 (24.09)	128.60 (24.27)	128.57 (25.55)	0.604
DBP (mmHg)	74.89 (14.57)	75.95 (16.01)	76.51 (16.77)	0.270
LVEF (%)	49.00 (38.50-58.00)	45.00 (36.00-55.00)	48.00 (40.00-56.00)	0.016
WBC (×10^9^/L)	5.14 (1.52)	6.22 (1.65)	9.53 (4.22)	<0.001
Neutrophil count(×10^9^/L)	3.00 (2.41-3.60)	4.16 (3.50-5.00)	6.72 (5.20-9.32)	<0.001
Monocyte count(×10^9^/L)	0.40 (0.30-0.50)	0.50 (0.40-0.60)	0.69 (0.50-0.90)	<0.001
Lymphocyte count(×10^9^/L)	1.40 (1.10-1.80)	1.10 (0.81-1.48)	0.71 (0.50-1.10)	<0.001
RBC (×10^12^/L)	4.11 (0.76)	4.07 (0.77)	4.06 (0.81)	0.607
HGB (g/L)	123.76 (21.57)	122.87 (23.26)	121.52 (23.68)	0.311
PLT (×10^9^/L)	152.00 (121.00-200.50)	167.00 (133.00-211.75)	169.00 (124.00-222.00)	<0.001
ALB (g/L)	36.67 (4.57)	35.36 (4.62)	33.67 (5.36)	<0.001
ALT (U/L)	20.00 (14.00-31.00)	20.00 (13.00-35.00)	23.00 (14.00-49.00)	<0.001
AST (U/L)	24.00 (19.00-34.00)	25.00 (19.00-35.00)	29.50 (20.00-52.00)	<0.001
Cr (umol/L)	77.00 (62.00-100.00)	87.00 (68.00-120.00)	104.00 (76.75-160.25)	<0.001
BUN (mmol/L)	6.40 (5.15-8.37)	7.20 (5.55-10.05)	9.14 (6.46-14.11)	<0.001
FPG (mmol/L)	5.20 (4.50-6.00)	5.30 (4.70-6.20)	5.60 (4.80-6.90)	<0.001
TG (mmol/L)	1.17 (0.87-1.56)	1.13 (0.89-1.52)	1.14 (0.90-1.58)	0.653
TC (mmol/L)	3.85 (3.13-4.50)	3.80 (3.16-4.42)	3.57 (3.02-4.23)	0.013
HDL-C (mmol/L)	1.03 (0.85-1.22)	1.00 (0.83-1.16)	0.92 (0.73-1.14)	<0.001
LDL-C (mmol/L)	2.32 (1.75-2.99)	2.31 (1.81-2.88)	2.20 (1.74-2.73)	0.079
NT-proBNP (pmol/L)	3170.00 (1895.00-4710.00)	3767.00 (2240.25-5832.75)	4193.00 (2298.50-6231.50)	<0.001
Anti-heart failure treatment				
Diuretic	461 (95.64%)	466 (96.68%)	464 (96.07%)	0.622
ACEI/ARB/ARNI	282 (58.51%)	309 (64.11%)	251 (51.97%)	<0.001
Beta-blockers	362 (75.10%)	387 (80.29%)	354 (73.29%)	0.030
Digitalis	192 (39.83%)	221 (45.85%)	244 (50.52%)	0.004
SGLT-2	55 (11.41%)	54 (11.20%)	53 (10.97%)	0.977

CHD, coronary heart disease; NYHA, New York Heart Association; LVEF, left ventricular ejection fraction; SBP, systolic blood pressure; DBP, diastolic blood pressure; TG, triglyceride; TC, total cholesterol; HDL-C, high-density lipoprotein cholesterol; LDL-C, low-density lipid cholesterol; Cr, creatinine; WBC, white blood cell count; RBC, red blood cell count; HGB, hemoglobin; PLT, platelet count; ALT, alanine aminotransferase; AST, aspartate aminotransferase; ALB, albumin; BUN, urea nitrogen; NT-proBNP, N-Terminal Pro-Brain Natriuretic Peptide; ACEI, angiotensin-converting enzyme inhibitors; ARB, angiotensin receptor inhibitors; ARNI, angiotensin receptor neprilysin inhibitors; SGLT-2, sodium-dependent glucose transporters 2.

### Follow-up outcomes

**Figure 2:**
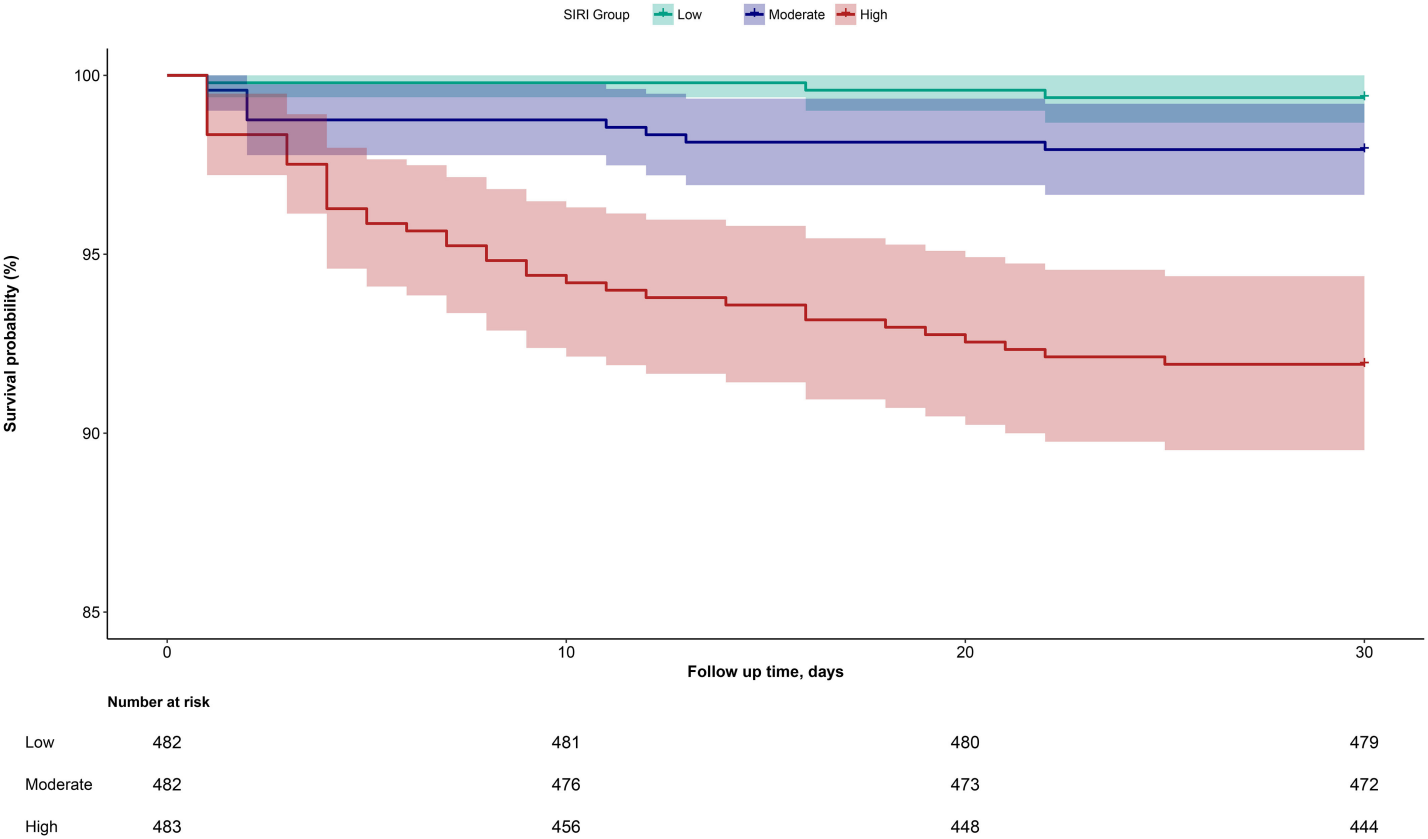
Cumulative survival rate curves of ADHF patients in SIRI group.

During the 30-day observation period, 53 deaths were recorded among the 1,448 ADHF patients. The mortality rates across the three SIRI groups were 0.62% (3 deaths), 2.07% (10 deaths), and 8.28% (40 deaths), respectively. **Figure 2** shows the 30-day survival curves according to SIRI group, indicating that the 30-day mortality rate was significantly higher in the high SIRI group compared to the low and medium SIRI groups (Log-rank *P* < 0.0001).

### Association between SIRI and 30-day mortality in ADHF patients

**Table 2** presents the adjusted HRs for all-cause mortality with SIRI treated as both a continuous and categorical variable. From Model 1 to Model 3, the HRs for the association between SIRI and 30-day mortality in ADHF patients gradually decreased (HR: 1.32 *vs*. 1.24 *vs*. 1.21), but the positive association remained consistent. In the final model, each standard deviation increase in SIRI was associated with a 21% increase in the 30-day mortality risk for ADHF patients (HR: 1.21, 95% CI: 1.02-1.44). Additionally, compared to ADHF patients with low SIRI, those with high SIRI had a 685% increased risk of 30-day mortality (HR: 7.85, 95% CI: 0.98-62.82). In all models, SIRI showed a significant positive trend with 30-day mortality in ADHF patients (All *P*-trend < 0.05).

**Table 2:** Multivariable Cox regression analysis of the association between SIRI and 30-day mortality in patients with ADHF.

	Hazard ratios (95% confidence interval)		
	Model 1	Model 2	Model 3
SIRI (Per SD increase)	1.32 (1.24, 1.41)	1.24 (1.14, 1.35)	1.21 (1.02, 1.44)
SIRI (tertiles)			
T1 (Low)	Ref	Ref	Ref
T2 (Moderate)	2.40 (0.65, 8.83)	2.22 (0.60, 8.26)	4.46 (0.53, 37.23)
T3 (High)	8.60 (2.62, 28.22)	8.35 (1.26, 15.03)	7.85 (0.98, 62.82)
*P*-trend	<0.0001	0.0078	0.0250

Model 1 adjusted for gender, age, hypertension, diabetes, stroke, CHD, NYHA classification, SBP, DBP.

Model 2 adjusted for model 1 + LVEF, NT-proBNP, Cr, FPG, Alb, RBC, PLT.

Model 3 adjust for: Model 2 + AST, TG, HDL-C, LDL-C and BUN.

**Figure 3:**
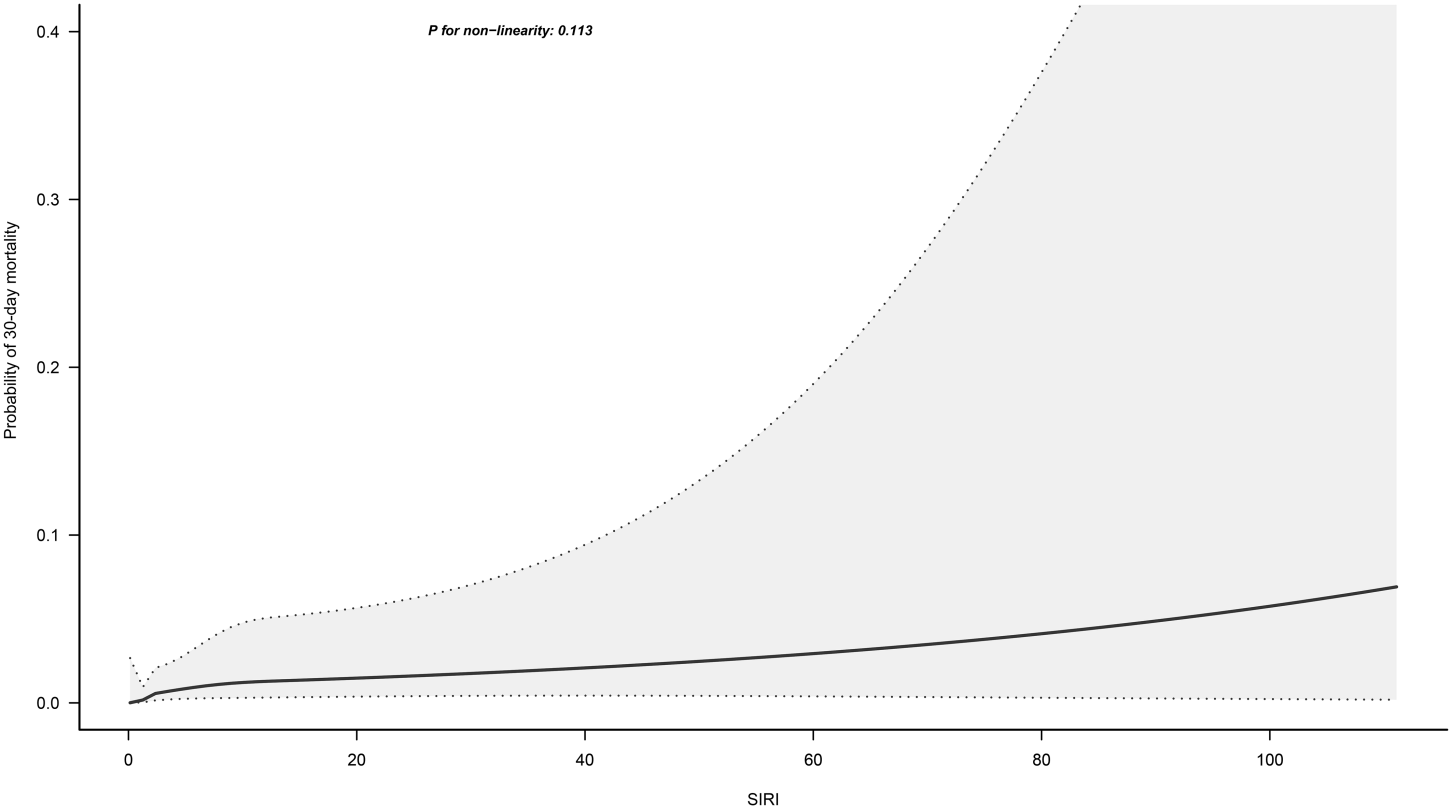
Fitting the dose-response relationship between SIRI and 30-Day Mortality in ADHF Patients with 4 knots restricted cubic spline. Adjusted for gender, age, hypertension, diabetes, stroke and CHD, NYHA classification, SBP, DBP, LVEF, NT-proBNP, Cr, FPG, Alb, RBC, PLT, AST, TG, HDL-C, LDL-C and BUN.

**Figure 3** illustrates the dose-response relationship between SIRI and 30-day mortality in ADHF patients, modeled using RCS with four knots. Overall, as SIRI increased, the 30-day mortality risk in ADHF patients rose linearly, showing a significant linear association (*P* for non-linearity = 0.113).

### Subgroup analysis

**Table 3** displays the results of stratified analyses based on age, gender, LVEF, NYHA classification, and comorbidities. Likelihood ratio tests for interactions between these factors and SIRI revealed no significant effect modifications on the association between SIRI and 30-day mortality in ADHF patients (All *P*-interaction > 0.05).

**Table 3:** Stratified analysis showed the relationship between SIRI and 30-day mortality in patients with ADHF in different age, gender, NYHA classification, LVEF and whether combined with hypertension/diabetes/cerebral stroke/CHD.

Subgroup	Adjusted HR (95%CI)	*P* for interaction
Age (years)		0.3960
18-64	1.69 (0.80, 3.59)	
65-100	1.17 (0.98, 1.40)	
Gender		0.9049
Male	1.20 (1.01, 1.44)	
Female	1.24 (0.77, 1.99)	
NYHA classification		0.2284
III	1.55 (1.02, 2.36)	
IV	1.17 (0.97, 1.41)	
LVEF		0.6167
HFrEF (LVEF < 40%)	1.12 (0.79, 1.58)	
HFmrEF (LVEF 40–49%)	0.83 (0.33, 2.09)	
HFpEF (LVEF ≥ 50%)	1.27 (1.03, 1.56)	
Hypertension		0.9122
Yes	1.22 (0.98, 1.52)	
No	1.19 (0.92, 1.55)	
Diabetes		0.4062
Yes	1.14 (0.89, 1.44)	
No	1.33 (1.01, 1.75)	
Stroke		0.6949
Yes	1.24 (1.01, 1.52	
No	1.15 (0.86, 1.55)	
CHD		0.6183
Yes	1.09 (0.69, 1.71)	
No	1.23 (1.02, 1.47)	

HFrEF, heart failure with reduced ejection fraction; HFmrEF, heart failure with mid-range ejection fraction; HFpEF, heart failure with preserved ejection fraction; other abbreviations as in **Table 1**.

Models adjusted for the same covariates as in model 3 (**Table 2**), except for the stratification variable.

### Predictive value of SIRI and its components for 30-day mortality

**Figure 4:**
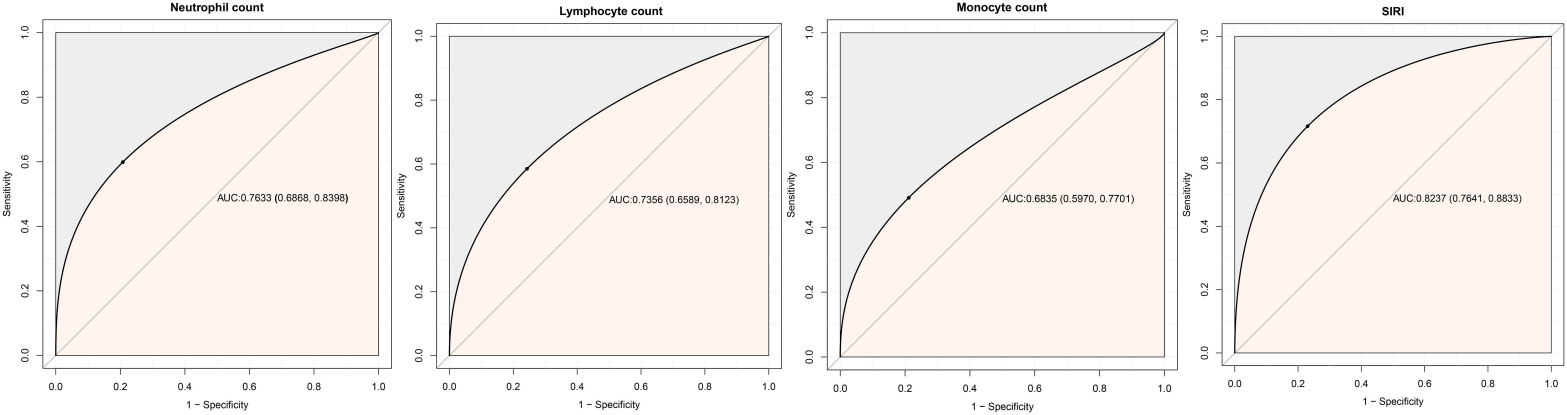
ROC analysis shows the predictive value of SIRI and its components on 30-day mortality in patients with ADHF.

The predictive value of SIRI and its components for 30-day mortality was shown in **Table 4** and **Figure 4**. The study found that SIRI had the highest AUC compared to neutrophil count, monocyte count, and lymphocyte count (AUC: neutrophil count 0.7633, monocyte count 0.6835, lymphocyte count 0.7356, SIRI 0.8237), significantly improving the prediction of 30-day mortality in ADHF patients (All DeLong *P* < 0.05). Additionally, the optimal threshold for SIRI in predicting 30-day mortality was calculated to be 7.3019, with a specificity of 0.9047 and a sensitivity of 0.6226.

**Table 4:** Area under the receiver operating characteristic curve of the SIRI and its components on 30-day mortality in patients with ADHF.

	AUC	95%CI low	95%CI upp	Best threshold	Specificity	Sensitivity
SIRI	0.8237	0.7641	0.8833	7.3019	0.9047	0.6226
Neutrophil count*	0.7633	0.6868	0.8398	6.5910	0.8215	0.6415
Lymphocyte count*	0.7356	0.6589	0.8123	0.7950	0.7563	0.6604
Monocyte count*	0.6835	0.5970	0.7701	0.5393	0.6136	0.7170

AUC, area under the curve; other abbreviations as in **Table ​1**. *P<0.001, compare with SIRI.

### Incremental predictive performance of SIRI and its components in mortality risk assessment

When SIRI was incorporated into the baseline risk model for predicting 30-day mortality, we observed a significant improvement in the model’s ability to predict death, with a C-index of 0.8921, up from 0.8695 (*P* value<0.01), and the net reclassification improvement and integrated discrimination improvement values of 0.16 (*P* value =0.02) and 0.09 (*P* value<0.01), respectively. These findings suggested that the inclusion of SIRI significantly enhances baseline risk models for predicting short-term mortality prognosis.

### Sensitivity analysis

Several sensitivity analyses were performed, and the results were consistent with the main findings (**Table 5**). Including the quadratic term for age did not materially alter the results. After excluding patients with multiple chronic conditions or those who died within the first three days, the results remained significant. Additionally, to mitigate the potential impact of missing data, the primary analyses were repeated using a complete covariate dataset constructed via the K-nearest neighbor interpolation method, yielding similar results. We repeated the primary analysis after excluding 182 patients with a history of chronic kidney disease or deep vein thrombosis or rheumatoid arthritis or periodontitis, and the results did not change substantially. Finally, we further adjusted diuretic, ACEI/ARB/ARNI, beta-blockers, digitalis, and SGTL-2 based on model 3, and the results remained robust.

**Table 5:** Sensitivity analysis of the association between SIRI and the risk of 30-day mortality in patients with ADHF.

	Hazard ratios (95% confidence interval)					
	Sensitivity-1	Sensitivity-2	Sensitivity-3	Sensitivity-4	Sensitivity-5	Sensitivity-6
SIRI (Per SD increase)	1.19 (1.01, 1.41)	1.23 (1.01, 1.50)	1.37 (1.10, 1.71)	1.22 (1.08~1.37)	1.19 (1.03, 1.37)	1.17 (1.02, 1.34)
SIRI (tertiles)						
T1(Low)	1(ref)	1(ref)	1(ref)	1(ref)	1(ref)	1(ref)
T2(Moderate)	4.75 (0.57, 39.41)	2.35 (0.23, 23.97)	inf. (0.00, Inf)	2.27 (0.61~8.45)	4.34 (0.45, 41.67)	5.31 (0.63, 44.58)
T3(High)	7.38 (0.92, 59.32)	8.59 (1.02, 72.74)	inf. (0.00, Inf)	4.74 (1.35~16.68)	7.48 (0.84, 66.80)	8.21 (1.03, 65.70)
*P*-trend	0.0377	0.0126	0.0171	0.005	0.0433	0.0280

inf: Infinity; Sensitivity-1: based on model 3, with additional adjustments for the quadratic term of age; Sensitivity-2: excluding patients with three chronic diseases at baseline; Sensitivity-3: excluding deaths occurring within the first three days of follow-up; Sensitivity-4: missing values were estimated using the K-nearest neighbor interpolation method; Sensitivity-5: Patients with a history of chronic kidney disease, deep vein thrombosis, rheumatoid arthritis, and periodontitis were excluded; Sensitivity-6: Further adjustments were made for treatment factors including diuretic, ACEI/ARB/ARNI, beta-blockers, digitalis, SGTL-2.

### Exploratory analysis: mediation effect of nutritional pathways on the association between SIRI and 30-Day mortality in ADHF patients

From the baseline characteristics, we observed that the high SIRI group had significantly lower levels of Alb, cholesterol, and lymphocyte count, suggesting a potential secondary deterioration in nutritional status among high SIRI patients. Based on this observation, we conducted an exploratory mediation analysis to assess the mediating role of nutritional pathways (Alb, TC, and lymphocyte count) in the association between SIRI and 30-day mortality in ADHF patients. The mediation analysis evaluated the indirect effects of these nutritional markers on the relationship between SIRI and 30-day mortality, and quantified the mediation effect by calculating the ratio of indirect effect to total effect.

The results (**Supplementary Table 5**) indicated that, except for lymphocyte count, both Alb and TC had significant indirect effects on the SIRI-related 30-day mortality in ADHF patients. Specifically, Alb accounted for approximately 24.46% of the mediation effect, while TC accounted for approximately 13.35%.

## Discussion

This cohort study based in Jiangxi, a city in southern China, demonstrated a significant linear positive correlation between SIRI and 30-day mortality in patients with ADHF. Additionally, compared to neutrophil count, monocyte count, and lymphocyte count, SIRI significantly improved the predictive value for 30-day mortality in ADHF patients. Overall, SIRI emerged as an important inflammatory marker for predicting short-term adverse outcomes in ADHF patients in southern China.

It is well-known that ADHF is one of the most common conditions requiring hospitalization among the elderly, frequently leading to severe adverse events shortly after onset, resulting in extremely poor outcomes (1–4). Activation of inflammatory pathways is one of the key mechanisms driving ADHF exacerbation. During ADHF episodes, multiple inflammatory mediators and cytokines are upregulated (4, 10–12, 52); further clinical evidence supports the early identification of inflammatory markers as crucial for assessing adverse outcomes associated with ADHF (53–56).

SIRI is a newly proposed systemic inflammation marker, calculated from neutrophil count, monocyte count, and lymphocyte count. Recent studies have provided evidence for the prognostic value of SIRI in HF patients (36–38). For example, a study by Ma et al. found that among ischemic HF patients undergoing PCI in Beijing, those with high SIRI had a 61% higher risk of death within three years compared to those with low SIRI (36). Another study in Beijing by Zhu et al. reported that high SIRI patients with chronic HF had a 138% increased risk of in-hospital mortality and a 39% higher long-term mortality risk compared to low SIRI patients (37). Additionally, a study based on the MIMIC database showed that high SIRI was associated with a 41% higher 90-day mortality risk and a 19% higher one-year mortality risk among elderly HF patients in the USA (38). In the present study, we found that ADHF patients with high SIRI had a 685% higher risk of 30-day mortality compared to those with low SIRI. Compared to similar studies, our research focuses on ADHF patients, and the evidence is more applicable to the southern Chinese population than the studies from northern China (36, 37). Notably, these studies suggest that high SIRI provides significant risk information for short-term adverse outcomes in HF patients.

The predictive value of SIRI for mortality has been widely discussed in recent years; Specifically in patients with oncological diseases the predictive accuracy of SIRI for survival ranges between 0.62 and 0.77 (57–61). In dialysis patients, the AUC for SIRI predicting adverse mortality events is 0.627 (62). In ischemic stroke patients, the predictive accuracy of SIRI for mortality is 0.63 (63). In acute myocardial infarction or chronic kidney disease patients, SIRI’s predictive accuracy is around 0.62 (26, 64). For aortic dissection patients, SIRI predicts in-hospital mortality with an accuracy of 0.72 (25). Furthermore, data from MIMIC indicates that combining SIRI with the Simplified Acute Physiology Score II predicts 90-day mortality in elderly HF patients with an accuracy of 0.656 (38). A study on chronic HF patients in Beijing reported an AUC of 0.6939 for in-hospital mortality prediction and 0.6182 for predicting three-year mortality (37). In the present study, we also evaluated the predictive performance of SIRI for 30-day mortality in ADHF patients, finding a strong predictive performance with an AUC of 0.8237. These results suggest that SIRI has predictive value for mortality in various diseases, especially in predicting short-term mortality in critical illnesses.

The mechanisms by which high SIRI leads to poor outcomes in ADHF patients remain unclear. However, the calculation method indicates that elevated neutrophil and monocyte counts, along with reduced lymphocyte counts, are the main contributors to high SIRI, a phenomenon also observed in the baseline characteristics of the current study (**Table 1**). From the perspective of immune cell interactions: (1) Neutrophils are primary participants in immune responses and can worsen HF by releasing peroxidase (65); furthermore, inflammatory cytokines released by neutrophils may induce lymphocyte apoptosis (66). Persistent high neutrophil and low lymphocyte counts in blood tests indicate severe myocardial damage and poor prognosis (67, 68). (2) Monocytes participate in the inflammatory response to myocardial injury, immune cell activation, myocardial cell hypertrophy and fibrosis, and apoptosis and necrosis (69); additionally, activated monocytes can release various inflammatory cytokines (70), potentially leading to lymphocyte apoptosis (66, 71). Overall, the direct link between high SIRI and poor outcomes in ADHF may involve secondary inflammatory cytokine release by neutrophils and monocytes and subsequent lymphocyte apoptosis. Besides, in the current study, we also considered that nutritional factors might mediate the association between high SIRI and mortality. The baseline characteristics show that patients with high SIRI have significantly lower Alb, cholesterol, and lymphocyte counts, indicating deteriorated nutritional status (72–74). To clarify whether nutritional factors mediate SIRI-related mortality, we conducted a mediation analysis, revealing that Alb and TC, besides lymphocyte count, significantly indirectly contributed to the 30-day mortality of ADHF patients related to high SIRI. This finding supports our hypothesis and provides reference material for the mechanisms leading to adverse events associated with SIRI.

For a long time, the high incidence of adverse prognosis in ADHF patients has been an important challenge for clinicians (1, 4, 7–9). Therefore, early identification of important factors affecting the deterioration of patients with ADHF is a very important task. In general, an ideal biomarker for prognostic assessment should be able to be used to independently assess and predict the risk of developing an adverse prognosis, allow for early and accurate risk stratification of patients, and provide information to guide treatment independent of etiology (75, 76). In the current study, we tested the value of a new inflammatory indicator in the assessment of short-term poor prognosis in patients with ADHF. Our results showed that SIRI was an independent risk factor for poor prognosis of short-term mortality in patients with ADHF and had a high predictive value for short-term mortality prognosis (AUC=0.8237). It should be noted that the assessment of SIRI is very simple and only requires routine blood measurements to be performed. Combined with the excellent predictive performance of SIRI in terms of short-term adverse prognosis in patients with ADHF and its important role in risk assessment for a wide range of chronic diseases (29–35), SIRI may have a good potential for generalized application to provide clinicians with useful information for risk assessment. Additionally, for the subsequent construction of predictive models for the poor prognosis of ADHF patients, we suggest that the inclusion of SIRI could be considered.

### Strengths and limitations

This study has several notable strengths stemming from the novelty of its findings and the characteristics of the study population: (1) SIRI shows considerable predictive value for 30-day mortality in ADHF patients (AUC: 0.8237). This is promising for ADHF patients, as SIRI is easily obtainable and effective. (2) To the best of our knowledge, this is the first study to evaluate the relationship between SIRI and short-term mortality in ADHF patients, with validation across multiple sensitivity analyses.

However, some potential limitations must be acknowledged: (1) The participants in this study were primarily from Jiangxi, a southern city in China, which may limit the generalizability of our findings to northern China or other racial and ethnic groups. (2) This study is non-interventional, so it cannot assess the impact of anti-inflammatory treatments on outcomes in hospitalized ADHF patients. (3) Our study mainly evaluated the predictive ability of SIRI at the time of admission for subsequent adverse events; the impact of the dynamic changes in SIRI during hospitalization on prognosis remains unclear and requires further investigation. (4) As with any observational study, residual confounding cannot be entirely eliminated. (5) The value of k in the KNN interpolation algorithm in the current study takes the value of 10, which may relatively lead to a degradation in the performance of the algorithm, which in turn affects the prediction of the results. (6) The current study was unable to assess the effect of time-varying confounders on study outcomes because repeated measures were not sufficiently available for the study population, and it is hoped that this limitation will be addressed in future studies.

## Conclusion

This cohort study based on a southern Chinese population reveals a significant linear positive correlation between SIRI and 30-day mortality in ADHF patients, highlighting its important predictive value. According to our findings, incorporating SIRI into the monitoring regimen for ADHF patients may be crucial for preventing further disease progression.

## Data Availability

The raw data supporting the conclusions of this article will be made available by the authors, without undue reservation.
