# Supplementary material for: The systemic inflammation response index as a significant predictor of short-term adverse outcomes in acute decompensated heart failure patients: a cohort study from Southern China
Source: Front Endocrinol (Lausanne). 2024 Dec 23;15:1444663. doi: 10.3389/fendo.2024.1444663 (PMC11700808; doi:10.3389/fendo.2024.1444663)
Supplement: Supplementary file 1 [file DataSheet1.docx]

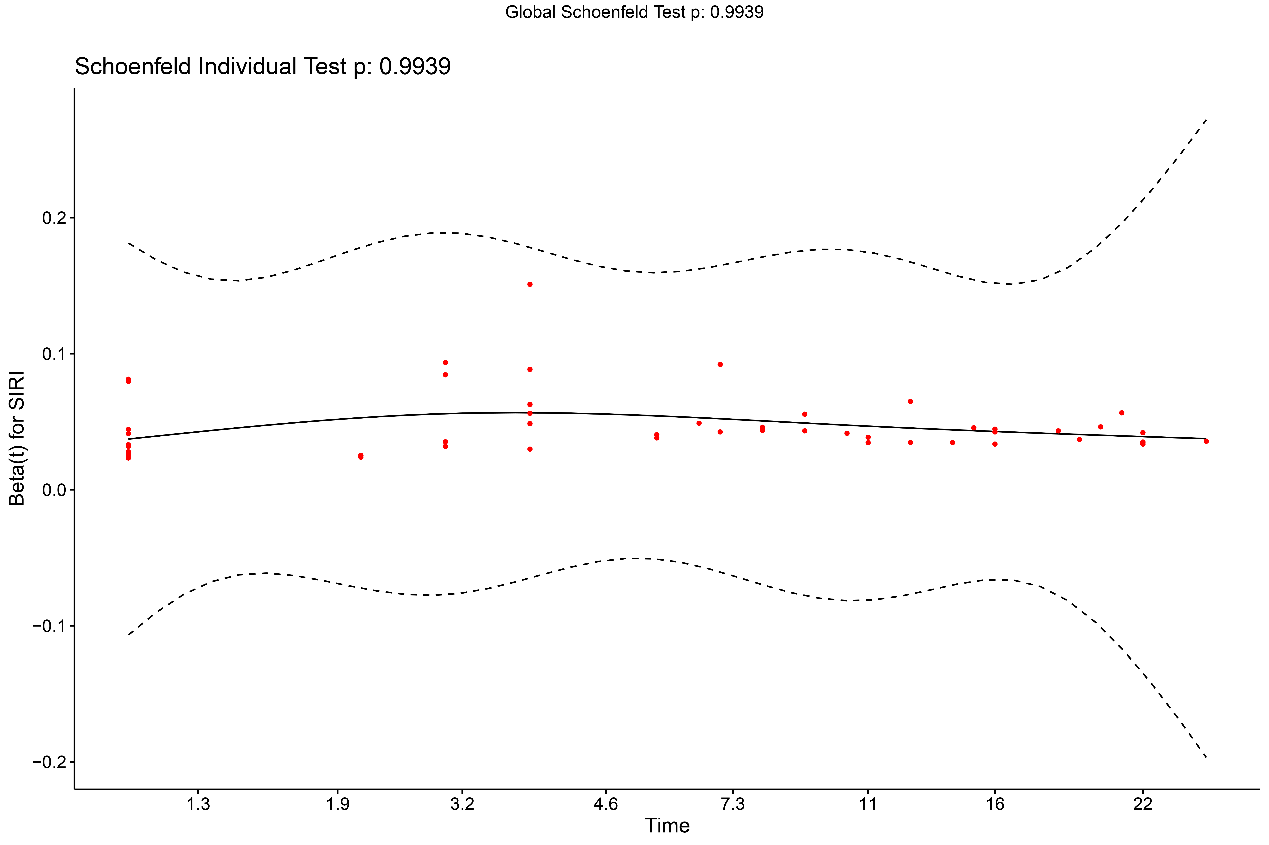


**Supplementary Figure 1**: Schoenfeld residual plot of SIRI over time with 30-day mortality in ADHF patients as the dependent variable. The p-value of Schoenfeld Residuals Test result is larger than 0.05 which indicated that SIRI is not a time dependent variable and can be analyzed by Cox Proportional Hazards Model.
